# Brain Tumor-Associated Psychosis and Spirituality—A Case Report

**DOI:** 10.3389/fpsyt.2017.00237

**Published:** 2017-11-14

**Authors:** Lars Levi Dutschke, Sarah Steinau, Roland Wiest, Sebastian Walther

**Affiliations:** ^1^University Hospital of Psychiatry, University of Bern, Bern, Switzerland; ^2^Department of Forensic Psychiatry, Psychiatric University Hospital Zurich, Zurich, Switzerland; ^3^Institute of Diagnostic and Interventional Neuroradiology, University of Bern, Inselspital, Bern, Switzerland

**Keywords:** psychopathology, psychosis, spirituality, auditory hallucinations, dysembryogenic neuroepithelial tumor, brain tumor

## Abstract

This case report describes a patient with a dysembryogenic neuroepithelial tumor localized in the posterior thalamus and internal capsule, which presented with psychosis including religiously determined severe self-mutilation, auditory hallucinations, and rituals. The patient’s history includes periodic religiousness over decades of her life suggesting that spirituality in this case might be a symptom of tumor progression. Our case reports on the topology-related effect of lesions on different brain networks involved in the phenomenology of the patient’s psychotic symptoms.

## Introduction

The location of brain lesions often correlates with specific functional deficits or neurological symptoms. The speed and duration of tumor growth may as well influence severity and course of symptoms. We will describe the case of a patient with a slowly growing brain tumor probably changing spirituality and religiousness periodically over decades in the patient’s life, eventually causing psychosis with severe self-injury conducted in belief of religious sacrifice and persistent auditory verbal hallucinations (AVH). Furthermore, we will put the symptoms into context with the regions affected by the lesion’s progress.

## Case Description

A 48-year-old woman without any significant past psychiatric history presented herself at the psychiatric emergency service at the University Hospital of Psychiatry Bern, Switzerland, in October 2015 with AVH and severe suicidal intent by self-harming with multiple thoracic stab wounds, which measured up to 7 cm in depth. She reported to have committed self-injuries due to religious sacrifice and a thorough communication with divine voices. AVH had first been noticed three years earlier in December 2012 and were considered to be “heavenly.” In the past, she had witnessed episodes of great spiritual interest and devotion starting at the age of 13 and reoccurring at ages 23, 32, and 41. During these episodes, she would join “Jehovah’s Witnesses” for 1–2 years and resign from them afterward because of a significant decrease of religiousness. Yet, she kept showing a higher-than-average devotion to spirituality.

Our patient was admitted to the inpatient department, where she presented a psychotic syndrome with grandiose (religious) delusions and extensive tension as well as a distinct feeling of blessedness. At the same time, she showed psychomotor retardation and blocking of formal thoughts. Ratings of affectivity—as assessed by the Bern Psychopathology Scale (1)—showed severe disinhibition in affectivity (Global Score Affectivity = +3) and moderate inhibition in the language and motor dimension (Global Score Language = −2; Global Score Motor behavior = −2). Additionally, [Positive and Negative Syndrome (PANSS) Scale (2)] scores showed moderate ratings in the positive subscore (29 points) and low levels of negative symptoms (17 points in the negative subscore).

According to the PSYRATS interview (3), AVH were frequent (>1/min). They occurred continuously in a normal speaking voice and were described as two different divine and persistently enjoyable voices, occasionally lasting for hours at a time. AVH were either imperative or in a dialog with the patient and generally of religious content (e.g., “In the name of Jehova, there is brother Agathon”).

## Brain Imaging

**Figure 1:**
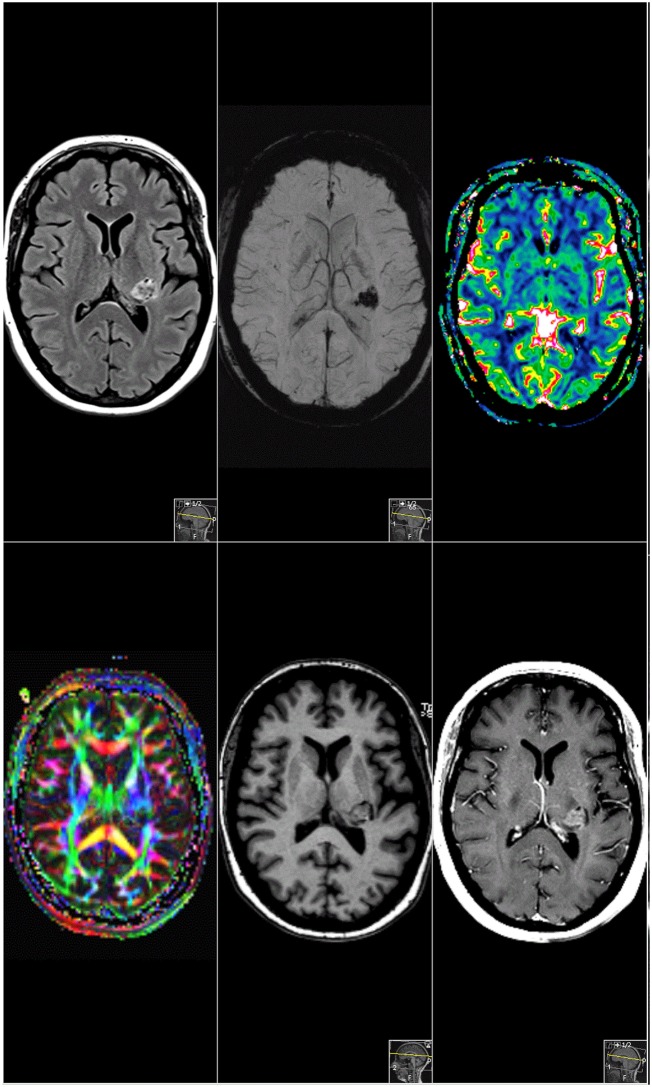
Magnetic resonance imaging. Sagittal cMRI reveals a supratentorial lesion involving the posterior limb of internal capsule, dorsolateral thalamus, partially, the posterior putamen and the corona radiata. The lesion enhanced inhomogenously after gadolinium administration in T1-weighted sequences.

Routine MRI was performed using T1w, T2w, FLAIR, SWI, and T1w CE sequences, extended by additional DTI and DSC perfusion imaging (see Figure 1). MRI revealed an ill-perfused ovoid lesion in the postero-lateral thalamus expanding into the posterior limb of the internal capsule and the posterior aspect of the putamen. The lesion appeared iso- to slightly hyperintense on T1w, with a mixed intensity on T2w/FLAIR, local susceptibility changes on SWI, and moderate Gadolinum-uptake on the T1w CE sequences. Diffusion Tensor Imaging indicated that the white matter pathways of the capsula interna were affected.

Brain imaging results suggested a low-grade glioma. Yet, cavernous malformations present similar features and need to be considered as a potential differential diagnosis.

## Course of Treatment

Initial psychopharmacological treatment with haloperidol (10 mg) and lorazepam (4 mg) demonstrated partial remission of psychotic symptoms. In the content of religious beliefs, a decrease of the intensity of the patient’s spirituality could be observed under treatment with a first-generation antipsychotic drug. Due to increased sleepiness, the medication was tapered and then changed from haloperidol to paliperidone (9 mg). Within the following 4–6 weeks of treatment, AVH decreased in frequency, but persisted. Consistently, PANSS ratings decreased both in the positive subscore (22 points) and in the negative subscore (9 points).

Physical and neurological examinations were unremarkable. The patient did not undergo any stereotactic external beam irradiation or surgical interventions due to a lack of tumor growth and neurological symptoms.

Follow-up cerebral MRI 3 months after diagnosis demonstrated no evidence of tumor progression. In the content of psychopathological symptoms, no aggravation of symptomatology was seen after discharge. Yet, the patient showed a slight increase of affective flattening with decreased facial expressiveness. Interestingly—under low-dose medication with paliperidone (less than 6 mg)—reoccurrence of clear above-average religious determination could be observed: Our patient described a renewed bond to God and concurrent concerns about her changing attitude toward religious worship after the antipsychotic medication had been tapered.

## Discussion

The present case describes a female patient reporting periodically reoccurring psychiatric symptoms since adolescence, who was treated in our department for AVH of divine voices and religious delusions leading to serious self-mutilation.

Routine MRI detected a brain tumor affecting the posterior thalamus, the posterior putamen, the dorsal internal capsule, and parts of the left external globus pallidus. The tumor is most likely a slowly growing dysembryogenic neuroepithelial tumor first occurring in adolescence. Its localization and affection of the tissue could be associated with the patient’s clinical symptoms. As in any lesion, there have certainly been alterations of the biochemical and spatial environment during growth or disintegrative periods, which might have resulted in a change of neural activity, most likely causing the above described symptoms. Nevertheless, we cannot exclude the possibility of a mere coincidental occurrence of the tumor entity and a psychotic disorder. Still, reports suggest that subjects who survived brain tumors in childhood and adolescence are at increased risk of developing psychoses (4).

Our case may stress the importance to comprehensively understand each symptom as the result of an imbalanced or disturbed network processing, distributing specific information in a complex of definable anatomical structures. Studies suggest the frontal, insular, anterior, and posterior cingulate cortices and most extensively the temporal lobe to be responsible for AVH (5–8). Schizophrenia symptoms have been associated with aberrant structure and function of the thalamus due to its critical role in cognition and perceptual networks (9). Indeed, schizophrenia is associated with structural alterations, e.g., a lower fiber count and altered functional connectivity of the thalamic midline regions. This particularly affects the circuits of the mediodorsal nucleus, projecting to the prefrontal cortex, the medial temporal lobe, and the reticular thalamic nucleus. Findings indicate an important role of the thalamus and its altered macro- and microstructural architecture in the pathophysiology of psychosis. A study by Anticevic and co-workers (8) confirmed thalamocortical dysconnectivity in schizophrenia across the mediodorsal nucleus and the lateral geniculate nucleus.

This patient’s tumor manipulates a very delicate region around the medial geniculate nucleus, which projects into the auditory cortex as part of the temporal lobe. Alterations of the auditory network in this part of the thalamus may have caused the reported AVH. Another symptom, which seems to be caused by malfunctioning of a processing loop in the affected region is the experience of transcendence and emotional affiliation leading to above-average spirituality and religiousness. The neurobiological correlates to the phenomenon of marked spirituality have previously been observed in temporal lobe epilepsy, which was linked to spiritual conversion (10). Interference with emotional (dys-)regulation has also been described previously and localized in the right ventral striatum, the head of the caudate, the left hippocampus, the dorsolateral prefrontal and orbitofrontal cortex, and bilateral thalamus (11). As the tumor affects some of these regions, it might also play a role in the observed emotional state influencing the development of strong spirituality in our patient. Functional brain imaging studies trying to identify brain regions involved in highly spiritual experiences suggest a multi-regional origin including activation of the caudate nucleus, globus pallidus, thalamus, and the inferior parietal lobe (12). Lesions of the inferior posterior parietal regions have also recently been found to potentially increase self-transcendence (13) creating a strongly spiritual experience. A recent study even demonstrated that both religiousness and spirituality may be manipulated through transcranial theta burst stimulation of the right inferior parietal lobe (14). In our patient, the lesion also affects the left thalamic ventral postero-lateral nucleus. This nucleus interferes with a network projecting into the somatosensory cortex in the parietal lobe causing alternatively processed self and body representation, which is also suggested to create self-transcendence as part of the experienced spirituality.

Additionally, we would like to discuss the peculiar observation that our patient had four strong spiritual episodes in a 9–10 years cycle starting at the age of 13 before hospitalization. Religiousness and spirituality could in this case be a nearly lifelong reoccurring symptom accompanying periodical brain tumor growth leading to a final psychotic episode and currently residing at a steady state as are the persisting symptoms (AVH and spirituality). We report a link between the localization of the lesion to specific networks, which may be involved in the creation of spiritual experiences. This observation may stimulate neurobiological studies exploring detailed aspects of human self-perception and conception. However, the main finding of this case is the impressive link between anatomical structures and very specific symptoms (religious delusion and AVH). Findings argue that aberrant connectivity in specific networks may lead to distinct symptoms in schizophrenia spectrum disorders [e.g., Ref. (15)], in contrast to a general dysconnectivity throughout the brain.

## Ethics Statement

Informed consent was obtained from the patient prior to publication. She consents to the use of her information for the purposes of publication of this case report.

## Author Contributions

LD and SS contributed equally to the design and concept of the work, data analysis and interpretation, drafting the article. SS collected the data. RW contributed expertise in neuroimaging and critical revision of the final manuscript. SW contributed to the design and concept of the work, the drafting of the article, and revision of the final manuscript.

## Conflict of Interest Statement

The authors declare that the research was conducted in the absence of any commercial or financial relationships that could be construed as a potential conflict of interest.

## References

[1] Strik W Wopfner A Horn H Koschorke P Razavi N Walther S The Bern psychopathology scale for the assessment of system-specific psychotic symptoms. Neuropsychobiology (2010) 61(4):197–209. 10.1159/000297737 20299814

[2] Kay SR Fiszbein A Opler LA The positive and negative syndrome scale (PANSS) for schizophrenia. Schizophr Bull (1987) 13(2):261–76. 10.1093/schbul/13.2.261 3616518

[3] Drake R Haddock G Tarrier N Bentall R Lewis S. The psychotic symptom rating scales (PSYRATS): their usefulness and properties in first episode psychosis. Schizophr Res (2007) 89(1):119–22. 10.1016/j.schres.2006.04.024 17095193

[4] Ross L Johansen C Dalton SO Mellemkjaer L Thomassen LH Mortensen PB Psychiatric hospitalizations among survivors of cancer in childhood or adolescence. N Engl J Med (2003) 349(7):650–7. 10.1056/NEJMoa022672 12917301

[5] Sun J Maller JJ Guo L Fitzgerald PB. Superior temporal gyrus volume change in schizophrenia: a review on region of interest volumetric studies. Brain Res Rev (2009) 61(1):14–32. 10.1016/j.brainresrev.2009.03.004 19348859

[6] Homan P Kindler J Hauf M Walther S Hubl D Dierks T. Repeated measurements of cerebral blood flow in the left superior temporal gyrus reveal tonic hyperactivity in patients with auditory verbal hallucinations: a possible trait marker. Front Hum Neurosci (2013) 7:304. 10.3389/fnhum.2013.00304 23805093 PMC3691504

[7] Kompus K Westerhausen R Hugdahl K The “paradoxical” engagement of the primary auditory cortex in patients with auditory verbal hallucinations: a meta-analysis of functional neuroimaging studies. Neuropsychologia (2011) 49(12):3361–9. 10.1016/j.neuropsychologia.2011.08.010 21872614

[8] Allen P Modinos G Hubl D Shields G Cachia A Jardri R Neuroimaging auditory hallucinations in schizophrenia: from neuroanatomy to neurochemistry and beyond. Schizophr Bull (2012) 38(4):695–703. 10.1093/schbul/sbs066 22535906 PMC3406523

[9] Pergola G Selvaggi P Trizio S Bertolino A Blasi G. The role of the thalamus in schizophrenia from a neuroimaging perspective. Neurosci Biobehav Rev (2015) 54:57–75. 10.1016/j.neubiorev.2015.01.013 25616183

[10] Hilger E Zimprich F Jung R Pataraia E Baumgartner C Bonelli S Postictal psychosis in temporal lobe epilepsy: a case-control study. Eur J Neurol (2013) 20(6):955–61. 10.1111/ene.12125 23663538

[11] Stegmayer K Horn H Federspiel A Razavi N Bracht T Laimböck K Ventral striatum gray matter density reduction in patients with schizophrenia and psychotic emotional dysregulation. Neuroimage Clin (2014) 4:232–9. 10.1016/j.nicl.2013.12.007 24455473 PMC3895617

[12] Beauregard M Paquette V. EEG activity in Carmelite nuns during a mystical experience. Neurosci Lett (2008) 444(1):1–4. 10.1016/j.neulet.2008.08.028 18721862

[13] Urgesi C Aglioti SM Skrap M Fabbro F. The spiritual brain: selective cortical lesions modulate human self-transcendence. Neuron (2010) 65(3):309–19. 10.1016/j.neuron.2010.01.026 20159445

[14] Crescentini C Di Bucchianico M Fabbro F Urgesi C. Excitatory stimulation of the right inferior parietal cortex lessens implicit religiousness/spirituality. Neuropsychologia (2015) 70:71–9. 10.1016/j.neuropsychologia.2015.02.016 25697502

[15] Walther S Stegmayer K Federspiel A Bohlhalter S Wiest R Viher PV. Aberrant hyperconnectivity in the motor system at rest is linked to motor abnormalities in schizophrenia spectrum disorders. Schizophr Bull (2017) 43(5):982–92. 10.1093/schbul/sbx091 28911049 PMC5581901

